# A probabilistic automated tagger to identify human-related publications

**DOI:** 10.1093/database/bay079

**Published:** 2018-09-13

**Authors:** Aaron M Cohen, Zackary O Dunivin, Neil R Smalheiser

**Affiliations:** 1 Department of Medical Informatics and Clinical Epidemiology, Oregon Health & Science University, Portland, OR, USA; 2 Department of Psychiatry, University of Illinois at Chicago, Chicago, IL, USA

## Abstract

The Medical Subject Heading ‘Humans’ is manually curated and indicates human-related studies within MEDLINE. However, newly published MEDLINE articles may take months to be indexed and non-MEDLINE articles lack consistent, transparent indexing of this feature. Therefore, for up to date and broad literature searches, there is a need for an independent automated system to identify whether a given publication is human-related, particularly when they lack Medical Subject Headings. One million MEDLINE records published in 1987–2014 were randomly selected. Text-based features from the title, abstract, author name and journal fields were extracted. A linear support vector machine was trained to estimate the probability that a given article should be indexed as Humans and was evaluated on records from 2015 to 2016. Overall accuracy was high: area under the receiver operating curve = 0.976, F1 = 95% relative to MeSH indexing. Manual review of cases of extreme disagreement with MEDLINE showed 73.5% agreement with the automated prediction. We have tagged all articles indexed in PubMed with predictive scores and have made the information publicly available at http://arrowsmith.psych.uic.edu/evidence_based_medicine/index.html. We have also made available a web-based interface to allow users to obtain predictive scores for non-MEDLINE articles. This will assist in the triage of clinical evidence for writing systematic reviews.

## Introduction

The MEDLINE database includes records of articles published in most of the high quality journals in biology and medicine. One unique feature is that all articles included in MEDLINE are read in full by doctor of philosophy-level curators who assign standardized indexing terms; in particular, Medical Subject Headings (MeSH) that represent the major topics discussed in the article (1). Over 60% of PubMed articles are indexed with the MeSH term *Humans*. These comprise a quite heterogeneous set of articles that includes studies of individual humans, human populations and studies that employ human cell lines or humantissues (including bodily fluids such as urine or serum). Nevertheless, for many purposes it is useful to know whether or not an article studies humans or human disease. Most systematic reviews, for example, only include evidence from randomized controlled trials from human, as opposed to animal, studies. Studying the progress of a therapy or treatment over time from early animal studies to standard human clinical care would also be aided by a *Humans* indexing term.

The high frequency of the MeSH *Humans* tag in MEDLINE entries presents an interesting and atypical situation for publication attribute tagging. Typically, the tags of interest, such as publication type tags, occur relatively infrequently, usually in <10% of articles. For example, the *Randomized Controlled Trial* publication type occurs in approximately 8% of MEDLINE indexed articles on humans (2). Other publication type tags, such as Cohort studies and Case–Control studies, occur even less frequently. One of the goals of the work presented here was to determine whether our prior approach for creating probabilistic taggers (2) would work well for such highly frequent publication attributes, especially given the heterogeneous mix of study types and topics involved.

The MeSH definition of the Humans term is very concise (https://www.ncbi.nlm.nih.gov/mesh/68006801): ‘Members of the species *Homo sapiens*’. This definition does not directly provide guidance on how to apply it to articles and other publications. Therefore, there is some vagueness in terms of what articles are sufficiently about ‘Members of the species *Homo sapiens’* to warrant the MeSH term and which are not. We will assume that human curation of the MeSH Humans indexing term is overall highly accurate (although we are not aware of any independent evaluation of its accuracy); however, as currently deployed the MeSH *Humans* terms has several limitations, which can be overcome by developing an automated tool that predicts a probability on whether or not a given article should be indexed under *Humans*.

First, tags are assigned in a binary manner, either present or absent. There is no scoring for confidence, uncertainty or specificity about humans. A probabilistic indexer, or tagger, estimates the probability that an article concerns *Humans*, as a number between 0 and 1, rather simply providing simple binary yes/no assignments. Since it is clear that many, if not most, MeSH terms are subject to inter-rater disagreements and variable levels of tagging inconsistency (e.g. (3)), a probabilistic measure of tag assignment is necessary to reflect these different levels of certainty. This is because yes/no indexing cannot handle borderline cases in which *Humans* does not appear to be applied consistently within MEDLINE. For example, only about half of articles indexed as Autobiography [Publication Type] are also indexed as *Humans* [MeSH]. About 17% of articles indexed as Health Policy [MeSH] lack *Humans* [MeSH] even though they discuss issues such as national healthcare reform. Another borderline case is quantitative models that employ data collected on humans (such as operations research analyses of how to optimally schedule patients in operating rooms). If viewed strictly, the indexing term *Humans* perhaps should not be applied to articles that study non-human entities; thus, current guidelines would not index as *Humans* studies of human genes or nucleotide sequences, agents that infect humans or animal models of human disease. Yet certainly these entities are studied in terms of the impact on human beings and one would like to give at least ‘partial credit’ to articles that are relevant to human health, so that these would be included in a looser or less strict search, but easily excluded from very strict searches.

We are unaware of similar automated systems that provide a probabilistic tagging human articles. However, the automated assignment of MeSH terms is a long-standing problem in biomedical informatics and library science, and there are several systems that attempt to annotate citations for all MeSH terms including Medical Text Indexer (MTI), DeepMeSH, MeSHLabeler, as well as many systems participating in the BIOASQ competition (4) (5) (6) (7). None of the systems or evaluations focused on the *Humans* tag specifically, and the evaluations focused on binary outcome measures such as F-measure, instead of probabilistic outcome measures such as (area under the receiver operating curve) AUC or Brier score (e.g. (8)). Since these approaches do not produce a probabilistic-interpretable tagging, they are difficult to directly compare with the probabilistic approach presented here that allows user-selected cut-offs, even when they do specifically assign human tag predictions. While the MTI system is in current use making suggestions to indexers at the National Library of Medicine, it is unclear whether the accuracy levels of the other systems are ready for generalized automated deployment. In general, these approaches are intended to suggest specific tags to reduce annotator workload. In contrast, probabilistic tags are intended to provide flexibility to literature searching and review, such as for systematic reviewers, guideline authors and other users surveying potentially large amounts of biomedical literature.

The probabilistic Humans tag developed here provides a directly interpretable numeric level of confidence that the tag is correctly assigned. For instance, a probability score of 0.99 means that of 100 articles receiving that score, 99 will be correctly tagged as *Humans* and one will be a false positive. A probability score of 0.001 can be interpreted as meaning that of 1000 articles receiving that score, 999 will be truly negative for the *Humans* tag, and one will be a false negative. Making a probabilistic tagger allows a user to decide for themselves what threshold of confidence is warranted for their own purposes—a narrow definition of human or a more relaxed definition. This is particularly valuable for systematic reviewers, who would like to utilize MeSH indexing but need high recall because they cannot afford to miss any potentially relevant publications. In this manner, the user, not the classification system, can apply a probability cut-off appropriate for their task, precision/recall requirements and workload.

Second, there is a variable, and sometimes, long delay between an article being published and being annotated for MeSH, including the *Humans* tag. It may take as long as 3 months or more for a newly published article to receive MeSH indexing terms, which creates a problem for searching and assessing the most recent literature. Users and use cases that require the latest published literature cannot make use of MeSH terms in their searches, since unannotated publications will be missed. For example, systematic review groups who are collecting all relevant published evidence on a given topic need to have comprehensive, up to date indexing of articles. For systematic review groups, and especially the emerging trend of ‘living systematic reviews’ (9) this imposes major limitations in search strategy and management of up-to-date literature. An automated probabilistic *Humans* tag can be generated directly from the bibliographic data and made available immediately. Applying the predictive model has a low per-article computational cost, and the probabilistic tag can be generated in advance as soon as an article is published for storage in a database or generated ‘on the fly’ when a user creates a collection of articles of interest.

Third, performing a comprehensive search of the literature requires searching databases beyond MEDLINE and PubMed. However, databases such as CINAHL (Cumulative Index to Nursing and Allied Health Literature), Embase (Excerpta Medica dataBASE) and PsycInfo have a ‘*Humans* type’ indexing tag, but without consistent, transparent criteria and so cannot be considered as interchangeable with MeSH terms. Combining and filtering the results of searches across databases require a consistent set of filtering criteria, no matter which database an article was originally found. An automated probabilistic *Humans* tag can provide useful, consistent and automated filtering criteria across databases.

For these reasons, a probabilistic *Humans* tag provides previously unavailable timeliness and flexibility to literature searching for systematic reviewers and other users desiring a more customizable search and filtering tool. Combining several filtering steps based on probabilistic tagging can provide additional user value. We have previously demonstrated the value of probabilistic tagging of articles that represent the *Randomized Controlled Trials* [Publication Type] (2) and extend the same general approach here to provide probabilistic estimates for *Humans* in PubMed articles. No MEDLINE-specific features are employed in the model, so that it can be used to tag newly published articles as well as those that are included in other bibliographic databases.

## Materials and methods

We carried out random sampling of MEDLINE records between the years 1987 and 2016. These records were separated into two data sets, a training set consisting of 1 077 268 records from the years 1987–2014, and a testing set consisting of 816 937 records from the years 2015 and 2016. This number of records was chosen as being ∼10% of annotated MEDLINE records with available abstracts from this period. Initial experimentation showed that this large training set should be enough to saturate learning on combinations of features we intended to use (2). All model building was performed on the training set, and the testing set used only to evaluate the final model.

The probabilistic tagger was built in Python (www.python.org) with a linear support vector machine (SVM) model, using the liblinear library (10). We have found in past research that this implementation of SVM performs well and is relatively fast. The liblinear SVM is able to handle the large number of training samples that were used to create the model presented here. We have had convergence issues with other implementations of SVM using similarly sized large training sets (2). In addition, as in our prior work, the modified Rüping method was employed to map the signed margin distances produced by the SVM into probabilities (11).

The citation database was pre-processed into a large number of extracted features per citation, which were stored in a PostgreSQL database (https://www.postgresql.org/). Features were generated by specifically written Python classes, and each Python class generates a set of related features for each citation. For example, title unigrams are one feature class, and title bigrams are another feature class.

Features that were extracted and evaluated for the probabilistic human tagger include the following:

- Title-based features: uni-, bi- and trigrams extracted from the title after tokenizing on whitespace and punctuation, converting to lower case and removing stop words. Stemming was not employed. Also, word count, punctuation symbol count and title numeric term count were extracted as feature classes.
- Abstract-based features: uni-, bi- and trigrams extracted from the abstract after removing stop words. Also, word count, punctuation symbol count and abstract numeric term count were extracted as feature classes.
- Bibliographic features: author names, author count, journal name, and page count were extracted.

Where a stop word list was employed, the list from Andrew McCallum’s Bag-Of-Words Library was used (http://www.cs.cmu.edu/∼mccallum/bow/). Note that word count, punctuation symbol count, title numeric term count and page count are features that are not investigated in our prior work. It was thought that these features may include some additional predictive value that could help distinguish human from not-human articles.

Because it is important that the human tagger be applicable both to non-MEDLINE records and to MEDLINE records prior to indexing, no MeSH terms or features derived from MEDLINE-specific data were used as predictive features in this work.

For training and evaluation, the assignment (or not) of the MEDLINE *Humans* MeSH term was used as the reference standard for the prediction variable, that is, whether or not the article was about humans. Overall frequency of the MEDLINE *Humans* term was 65% on the training data set and 70% on the test data set.

Feature sets were evaluated both individually and in combination using the forward selection process, as described in our previous work (2). Briefly, starting with no feature sets included, each remaining feature set is evaluated in combination with currently included feature sets. At each stage, the feature set resulting in the highest performance gain is selected to be one of the currently included feature sets. The process iterates until no remaining feature set improves overall performance. In our previous work, we have compared this forward selection process with individual feature selection based on statistical criteria, such as *χ*^2^. The forward selection process has resulted in vastly superior models. Individual feature selection tends to throw out too many features that have weakly, but non-zero predictive value. In combination, these weakly predictive features provide a large incremental value that individual feature selection loses.

Five iterations of two-way cross-validation were used with the training data to evaluate each stage of the forward selection process. AUC (Area Under the receiver operating Curve) and the Mathews Correlation Coefficient (MCC) were used to evaluate the performance of each combination of features. At each iteration, the feature set, which gave the largest improvement in AUC, and if no change in AUC, the largest improvement in MCC, was chosen. At no point in the selection process was either AUC or MCC allowed to decrease.

Once the forward selection process was completed, the final model was trained on the entire training data set. This final model was then used to create probabilistic tags on the test set, and these tags were evaluated in several ways. First, the test set tags were evaluated against the MEDLINE *Humans* assignment for correctness. Secondly, we evaluated the distribution of tag probabilities across the MEDLINE Human positive and negative subsets of the test data. Finally, we manually examined and reviewed the extreme disagreements between the probabilistic tagger and the MEDLINE assignments. One hundred random cases were selected, where the Human MeSH tag was assigned and the probabilistic tagger predicted a tag probability of <0.01. Another 100 random cases were selected, where the Human MeSH tag was NOT assigned and the probabilistic tagger predicted a tag probability of >0.99. The cases were manually reviewed in a blinded manner applying the definition of the *Humans* MeSH term, and the results of the manual review were compared to the MeSH assignment as well as the probabilistic tagger prediction. Specifically, an article is marked as ‘human’ if it deals with human individuals, human populations or human-derived tissues (including bodily fluids or cells grown in culture). The blinded reviewer was allowed to look at the title, abstract, journal name and author list for the citation, as well as to read the full text article of the paper if deemed necessary. The reviewer did not have access to the assigned MeSH terms, MEDLINE publication type or probabilistic tagger prediction. The blinded reviewer was instructed to mark each article as HUMAN, NOT_HUMAN or UNCERTAIN.

## Results

The forward selection process resulted in the final feature set shown in Table 1, along with the level of AUC and MCC performance achieved at each stage. Using these features, the final model performance on the test data set for several metrics is shown in Table 2, along with the cross-validation estimates based on the training data. Note that we present the binary outcome measures MCC, F1, Precision, Recall and Error Rate, here, as well as the probabilistic outcome measures AUC, and Brier Score. A default threshold of 0.50 was used to binarize the predictions. These binary outcome measures do not reflect the intended flexible use cases for the probabilistic tags and are included here only for comparison with prior work.

**Table 1:** Forward selection process results, showing best performing feature included at each stage using 5 × 2 cross-validation on the training data set

Stage	Feature	AUC	MCC
1	Abstract Bigrams	0.955	0.771
2	Abstract Unigrams	0.967	0.813
3	Journal Name	0.969	0.823
4	Title Unigrams	0.972	0.831
5	Title Bigrams	0.973	0.834
6	Abstract Trigrams	0.974	0.837

**Table 2:** Comparison of performance results predicted by cross-validation and actual results predicted on the test data set

Dataset	AUC	MCC	F1	Recall	Precision	Brier Score	Error Rate
Training	0.975	0.841	0.944	0.940	0.949	0.059	0.073
Test	0.976	0.833	0.950	0.946	0.955	0.056	0.070

The new features investigated (word count, punctuation symbol count, title numeric term count and page count) were not found to improve the final predictive model. However, even without these new features providing additional value, the model is highly accurate and demonstrates that the approach used to create the prior RCT (Randomized Controlled Trial) tagger also works well for the *Humans* tag assignment.

**Figure 1:**
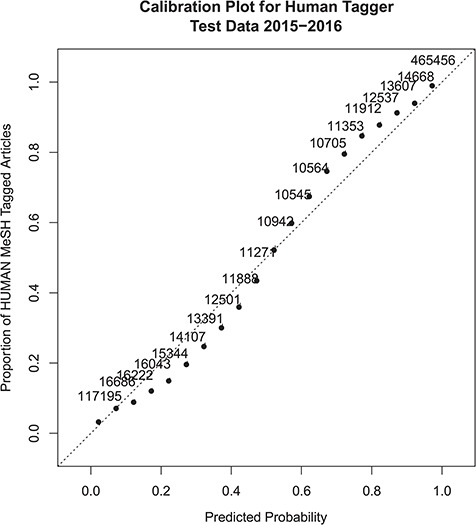
Probabilistic tagger confidence score calibration plot. The x-axis represents the predicted probability score, and the y-axis shows the proportion of articles within a similar probability score range that were assigned the *Humans* MeSH term. Numbers next to the dots show the number of samples included in the probability score range used to calculate the MeSH *Humans* proportion. The dotted line x = y shows perfect calibration for comparison.

Overall the performance figures on the test data set correlate very closely with the cross-validation estimates. Calibration of the model to the actual *Humans* MeSH assignment was very good, as shown in Figure 1. Figure 1 is a calibration plot of the probability predictions on the test data set, as compared to the overall proportion of MeSH *Humans* assignments for articles at each level of predicted probability. The plot shows that the model slightly over-predicts the proportion for probability values between 0.10 and 0.50 and slightly under-predicts the proportion for probability scores between 0.50 and 0.90. Calibration is almost perfect at the extremes. There appears to be no overall bias in the calibration as the under and over predictions are about the same. The adjusted *R*^2^ statistic between the predictions and the actual MeSH Human tag proportions is 0.982.

**Figure 2:**
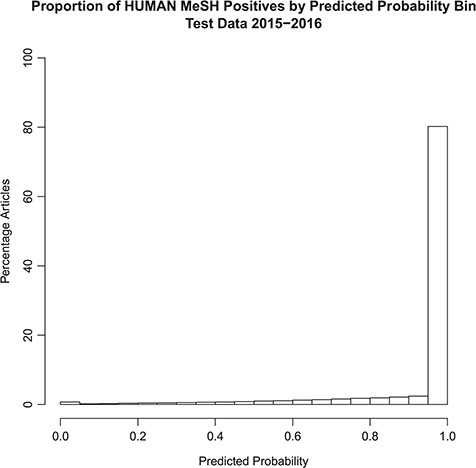
Probabilistic tagger predicted probability score distribution over articles in the test set, consisting of articles published in 2015–2016 and assigned the Humans MeSH term. Shows the distribution of the probability estimates of these articles as predicted by our model versus the percentage of articles in the test set assigned the MeSH Humans term.

**Figure 3:**
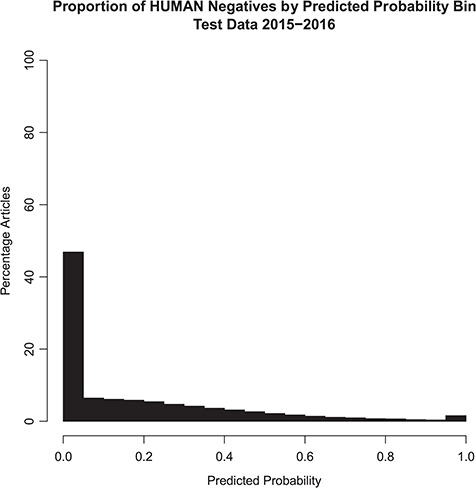
Probabilistic tagger predicted probability score distribution over articles in the test set, consisting of articles published in 2015–2016 and NOT assigned the Humans MeSH term. Shows the distribution of the probability estimates of these articles as predicted by our model versus the percentage of articles in the test set NOT assigned the MeSH Humans term.

The distribution of the model probability estimates for the human tagger for the test set articles with and without the *Humans* MeSH term is shown in Figures 2 and 3, respectively. Figure 2 shows that the vast majority of articles that are assigned the MeSH *Humans* term are scored very highly by the tagger, typically >0.95, with extremely few of these articles scored <0.50. Figure 3 shows that the vast majority of articles that are NOT assigned the MeSH *Humans* term are scored very low by the tagger, typically <0.10, with a monotonically decreasing amount assigned between 0.10 and 0.90. Interestingly, there is a small increase in negative MeSH *Humans* articles scored >0.90. Looking closely at Figure 2, there is also a very small bump on positive MeSH articles scoring <0.10.

The ‘extreme disagreements’ between the tagger and the assigned MeSH tags were manually reviewed. One hundred articles were randomly chosen in which articles lacking *Humans* MeSH were scored >0.99 by our model; these samples represent the ‘bump’ in the histogram near 1.0 in Figure 3. In addition, 100 articles, which received *Humans* MeSH indexing but to which our model gave scores <0.01, were randomly chosen. These articles represent the small ‘bump’ in the histogram near 0.0 in Figure 2. When the model gave low predictive scores (<0.01), the manual reviewer agreed with the model 97% of the time. On the other hand, when the tagger predicted high scores (>0.99) in articles lacking *Humans* MeSH, the manual reviewer only agreed with the model 50% of the time. Overall, manual review agreed with the model in 73.5% of cases. Therefore, according to the manual review, when the model gives an article a low predictive score, the article is almost certainly not about humans. However, in some cases a high score will be assigned to articles that are in fact not about humans. This is consistent with the probabilistic interpretation of the tag. See Table 3.

**Table 3:** Comparison of manual review for cases of extreme disagreement between the MEDLINE assigned *Humans* MeSH term and the model’s predictive probability scores. One hundred cases of extreme prediction disagreement were selected randomly from articles with the MEDLINE *Humans* assignment but predictive tagger probabilities <0.01, and another 100 cases lacking the MEDLINE *Humans* term but having predictive tagger probabilities >0.99

	Manual review			
Disagreement type	Humans	Not Humans	Uncertain	Totals
Humans MeSH term assigned, tagger probability score < 0.01	2	97	1	100
Humans MeSH term not assigned, tagger probability score > 0.99	50	41	9	100
Totals	52	138	10	200

## Discussion

The probabilistic automated tagger performs with very high accuracy and calibration and gives similar results as that of MEDLINE curators overall. The high frequency and heterogeneous nature of human-related articles proved not to be a substantial problem for our machine-learning method. The differences between estimated cross-validation performance on the training set and performance on the held out test set were small and approximately evenly distributed in direction. Article metadata features alone were enough to reach high performance.

The majority of the tagger scores are quite binary, either <0.05 or >0.95. Still, a substantial fraction of articles fall into the middle range. Almost 50% of articles in the test set that do not have the Humans MeSH term score between 0.05 and 0.95. For articles that do have the Humans MeSH term assigned, the proportion of articles in the middle is less, but still notably ∼20%. These did appear to represent cases that were borderline for some reason (e.g. a review of animal models of human disease with relevance for potential treatments in man). It is important to provide the user with customizable tools in order to handle these articles in a manner appropriate for their specific use case. No binary tag assignment tool can offer a similar level of flexibility.

As a simple example, consider a researcher looking for narrative articles about humans in PubMed. One publication type of interest would be *Autobiographies*, which logically should also have the *Humans* MeSH term. A recent PubMed search found 3399 articles indexed as Autobiography. Of these, only 1376 also have the *Humans* MeSH term. The *Autobiographies* lacking the Humans MeSH term include seemingly obvious human-related articles such as ‘An interview with Claudio Stern’ (12), ‘Laura Frontali-my life with yeast’ (13) and ‘Autobiography of J. Andrew McCammon’ (14). These articles are scored 0.31, 0.19 and 0.40, respectively, with the probabilistic Human tagger. Perhaps an interview is less about Humans than about a specific human. Also, perhaps a ‘life with yeast’ may be more about yeast than about humans. Certainly there is some gray area and perhaps inconsistency about what constitutes a human article. A user searching for Autobiographies and limiting the search to articles having the Humans MeSH term would miss half the autobiographies. Removing the Humans MeSH term requirement would include 47 articles tagged with the MeSH term Bacteria and not the MeSH term Humans. The user would have no fine-grained controllable search options to address this problem. With a probabilistic tagger, a threshold of 0.10 would pick up all three of these example biographies as human articles. For a user requiring a much stricter definition, perhaps Autobiographies that are about the personal lives of the human beings instead of their work, a threshold of 0.50 would exclude all of the three example biographies. This underscores the need for flexible tools, customizable to different use cases.

We examined cases of extreme disagreement between the MEDLINE MeSH assignment and the model’s predictive scores. An independent blinded human expert found that when the model predicted NON-HUMAN, the article was almost always NON-HUMAN. However, when the MeSH term was not assigned but the model predicted HUMAN with high confidence, the expert agreed with the model only in half the articles. This suggests that a user who employs the model to retrieve human-related articles may safely discard articles having predictive scores below 0.01. The probabilistic nature of the tag along with the good level of calibration ensures that there will be a controllable proportion of false positives and false negatives at any chosen probability threshold. Since the threshold is customizable by the user for their specific purposes, the impact of these false positives and false negatives on workload should be small.

While the main results for the Humans tagger presented here are the probabilistic tagger evaluation measures of AUC and Brier score, the binary outcome measures are also highly accurate and compare favorably with prior binary label prediction work. The F1 measure on the test set here is 0.950. Yepes reported a maximum F1 of 0.9337 for the MeSH *Humans* term (8).

The Human probabilistic tagger is being used by our team to assign predictive scores to all articles indexed in PubMed, including newly published articles, and have been made public for download on our project website (http://arrowsmith.psych.uic.edu). As well, the model is used to assign predictive scores to articles that are retrieved through Metta (15), which carries out a unified high recall, de-duplicated retrieval of records not only from PubMed but also from EMBASE, CINAHL Plus, PsycINFO and the Cochrane Central Register of Controlled Trials. The Humans probabilistic tag will be supplemented by RCT predictive scores (2) and other automated publication type, study design and attribute taggers that are currently under development.

## Conclusion

The predictive model described here was highly accurate as evaluated by both a large-scale comparison with MEDLINE as well as manual expert review, achieving accuracy comparable to that of MeSH indexing itself. We have tagged with our predictive scores all articles in PubMed from 1987 through 2017 and are tagging newly published articles weekly as they appear. Using our automated tagging approach, most of these new articles will be tagged by our Humans probabilistic model prior to review for annotation by the MEDLINE indexers. The current database of articles tagged with the Humans probabilistic model is available at http://arrowsmith.psych.uic.edu/evidence_based_medicine/index.html.

This information will assist in the triage of clinical evidence during the initial phase of writing systematic reviews and also help ensure that the update process has ready access to the latest published articles.

## Notes

**Database URL**: http://clingen.igib.res.in/sage.
